# Knowledge, attitudes, and practices regarding avian influenza among poultry farmers near migratory bird habitats in Guidong County, China

**DOI:** 10.3389/fpubh.2025.1618292

**Published:** 2025-06-17

**Authors:** Yan Jiang, DaiJun Zhong, Yang Han, Yong Zhou, HaiJian Zhou

**Affiliations:** ^1^Department of Epidemiology and Health Statistics, School of Public Health, Xiangnan University, Chenzhou, China; ^2^Department of Parasitic and Vector-borne Disease Control, Changjiang County Center for Disease Control and Prevention, Changjiang, China; ^3^Institute for Infectious Disease Prevention and Control, Chinese Center for Disease Control and Prevention, Beijing, China

**Keywords:** migratory birds, avian influenza, knowledge-attitudes-practices, health education, structural equation modeling

## Abstract

**Objective:**

To assess the knowledge, attitudes, and preventive behaviors (KAP) regarding avian influenza among poultry farmers living near migratory bird habitats in Guidong County, China, and to identify determinants of these practices using structural equation modeling.

**Methods:**

A cross-sectional survey was conducted in July 2021 among 221 poultry farmers from three towns adjacent to migratory bird habitats. A structured questionnaire was used to collect data on KAP related to avian influenza. Descriptive statistics was used to analyze KAP levels. A structural equation model was developed with AMOS 24.0 to examine the relationships among knowledge, attitudes, and behaviors.

**Results:**

The overall mean KAP score was 32.97 ± 7.95 (51.5%) of maximum possible score, indicating suboptimal levels. In the fitted model, knowledge exerted both a direct effect on preventive behaviors (standardized path coefficient = 0.183) and an indirect effect mediated through attitudes (0.056). Attitude additionally influenced behavior directly (0.181). Goodness-of-fit indices confirmed robust model fit.

**Conclusion:**

Study findings indicate that poultry farmers living near migratory bird habitats in Guidong County demonstrate insufficient avian influenza–related knowledge, attitudes, and practices. Targeted health education that enhances accurate knowledge and fosters positive attitudes is critical to strengthening preventive behaviors and mitigating transmission risk.

## Introduction

Zoonotic diseases have increasingly commanded global attention over the past four decades, with studies indicating that approximately 60% of emerging infectious diseases originate from animals and around 70% are linked to wildlife reservoirs (1). Among these pathogens, avian influenza is particularly concerning in the 21st century due to its ability to cause severe respiratory infections and its potential to mutate via antigenic drift or shift—processes that not only precipitate outbreaks in domestic poultry but also raise the risk of interspecies transmission (2, 3). These events have profound implications for both human health and economic stability worldwide (4).

Migratory birds are recognized as natural reservoirs of avian influenza viruses and play a crucial role in their dissemination (5). Since these birds generally do not exhibit any symptoms during migration, they can both carry and spread the virus, thereby complicating detection efforts. When the virus contaminates water sources in their habitats, it becomes highly likely to infect nearby domestic poultry, establishing a transmission chain at the regional level (6). Moreover, the high variability of avian influenza viruses raises the potential for cross-species transmission to humans, which can ultimately lead to influenza outbreaks (7). Although the transmission of avian influenza viruses from birds to humans is rare, documented cases indicate that certain subtypes like H5N1, H5N8, H7N9, and H9N2 can infect humans and even facilitate human-to-human spread (8, 9). For example, migratory birds spreading the H5 influenza virus, causing three waves of influenza outbreaks across various regions (1, 3). Since 2003, several H5N1 outbreaks in South Korea have been linked to migratory birds (10). Additionally, between 2018 and 2020, 19 strains of the H7N7 avian influenza virus were identified in migratory birds in eastern China (11). The avian influenza viruses carried by migratory birds pose a significant threat to the poultry industry along their migration routes.

China lies along three major migratory flyways, posing unique challenges for avian disease prevention and control, especially in regions where poultry rearing is ubiquitous (12). To address this, we focus on Guidong County, Hunan Province. (The geographical coordinates are 113°37′ east longitude—114°14′, 25°44′ —26°13′ north latitude) —located along a central migratory flyway known as the “Millennium Bird Passage”—exacerbates the challenges of preventing and controlling avian diseases. Here, nearly every household engages in poultry rearing, and the close proximity of wild birds to domestic flocks increases the risk of virus recombination and potential transmission to humans (13–15). Although no major outbreaks have been reported in Guidong County to date, sporadic human infections (e.g., cases involving H3N8 in nearby cities) and genetic evidence suggesting mixed infections from wild and domestic birds underscore the latent risk (16, 17). Despite these risks, no studies have systematically investigated prevention behaviors in such ecologically vulnerable populations—a gap this study aims to fill.

The Knowledge-Attitude-Practice (KAP) model provides a robust framework for understanding health behavior determinants, proposing that individuals first acquire knowledge, which shapes their attitudes and beliefs, and ultimately drives practice (18). Numerous studies have investigated KAP regarding avian influenza prevention among general populations and poultry-related occupational groups in countries such as China, Nigeria, and India (18–22). For example, during five outbreaks in China between 2013 and 2017, 91 of 695 infected cases involved occupational poultry contact (20), yet many live-bird market workers still underestimate their risk and rarely adopt recommended preventive measures (23). Despite sustained efforts by Chinese authorities—including health education campaigns, market closures, and mandatory culling of infected flocks, the persistence of noncompliance among some farmers has limited these measures’ effectiveness (24, 25). Systematically employing the KAP (Knowledge, Attitude, and Practice) framework in avian influenza research, we explicitly test three hypotheses: (1) Knowledge-Action Disconnect: High-risk poultry farmers exhibit poorer KAP compliance compared to general occupational groups, despite comparable avian influenza knowledge levels. (2) Geographic Determinants: Proximity to migratory corridors directly correlates with risk perception and protective behavior adoption. (3) Attitude Mediation: Attitudes mediate the knowledge-to-practice relationship, explaining persistent prevention gaps.

To test these hypotheses, we implemented a cross-sectional study in Guidong County (July 2021) employing structural equation modeling to decode latent relationships between avian influenza knowledge, attitudes, and protective behaviors. This methodology extends conventional KAP research through dual innovations: first, by targeting high-risk populations at the human-wildlife interface; second, by enabling quantitative prioritization of modifiable behavioral factors—providing evidence-based guidance for optimizing prevention protocols in ecologically sensitive zones.

## Methods

### Study design and participants

This study employed a cross-sectional design and adhered to the STROBE (Strengthening the Reporting of Observational Studies in Epidemiology) guidelines for reporting observational research. The sample size was determined empirically, following the standard practice of including 5 to 10 times the number of influencing factors under investigation. In July 2021, we conducted a survey among 231 poultry farmers residing in Pule Town, Shatian Town, and Oujiang Town—key migratory bird stopover sites in Guidong County, China. Inclusion criteria for participants were: Poultry farmers who had settled in Guidong County’s migratory bird habitats for at least 1 year; Individuals who provided informed consent and voluntarily participated in the survey.

This study received ethical approval from the Ethics Committee of Xiangnan University (K2024-009-01). All participants provided verbal informed consent prior to their involvement.

### Instrument development and validation

The survey instrument was developed through a systematic process involving three key stages: (1) initial questionnaire construction based on comprehensive literature review and expert consultation, (2) pilot testing with 30 target population representatives, and (3) iterative refinement to establish the final version.

The structured questionnaire comprised four validated dimensions:

Demographic characteristics (19 items): Capturing age, gender, occupational classification, and socioeconomic indicators.

Avian influenza knowledge (12 items): Assessing recognition of Clinical manifestations (e.g., febrile respiratory symptoms); Transmission pathways (contact with infected poultry/excreta/migratory birds).

Risk perception attitudes (11 items): Evaluating perceived susceptibility and outbreak concern level.

Protective behaviors (20 items): Documenting PPE usage frequency (masks, aprons, waterproof footwear) during poultry handling operations.

In terms of scoring, for the knowledge section, 1 point was assigned for “yes” responses, and 0 points for “no” or “unclear” responses. A higher score indicated a higher level of knowledge. For the attitude section, Responses indicating a positive attitude were assigned 2 points, neutral responses received 1 point, and negative responses were given 0 points. The behavior section was assessed using a 4-point Likert scale, where “always” was assigned 4 points, “often” 3 points, “rarely” 2 points, and “never” 1 point.

The reliability and validity of the questionnaire were assessed, yielding an overall Cronbach’s *α* coefficient of 0.865, indicating good internal consistency. Specifically, the Cronbach’s α coefficient was 0.893 for the avian influenza-related knowledge dimension, 0.710 for the attitude dimension, and 0.762 for the behavior dimension, further supporting the questionnaire’s reliability. Additionally, the overall Kaiser-Meyer-Olkin (KMO) measure was 0.825, with values of 0.896 for the knowledge dimension, 0.699 for the attitude dimension, and 0.784 for the behavior dimension. Bartlett’s test of sphericity yielded *p*-values of less than 0.001 across all dimensions, confirming the questionnaire’s good construct validity.

The different dimensions were analyzed using maximum and minimum values, mean ± standard deviation, and scoring rate. The scoring rate was determined using the formula: Scoring rate = (Actual score of the overall questionnaire or dimension / Possible maximum score of the overall questionnaire or dimension) × 100%. Based on the scoring rate, scores below 60% were categorized as poor, 60–80% as moderate, and above 80% as good. This classification system was applied to evaluate the knowledge, attitudes, and practices related to avian influenza among poultry farmers in the migratory bird habitats surrounding Guidong County.

### Quality control

This study utilized door-to-door household surveys, conducting one-on-one interviews with participants in their residences. After obtaining verbal informed consent from participants, the surveys were distributed and completed under standardized protocols. All surveyors completed a training program including questionnaire administration simulations and role-playing exercises to ensure consistent interpretation of items. For individuals with literacy difficulties, trained surveyors provided assistance by reading each item aloud in a neutral and non-suggestive manner. Upon completion, researchers conducted an on-site review to check for completeness and accuracy before collecting the surveys.

### Statistical analysis

Data were entered into Excel and analyzed using SPSS 25.0 Qualitative data were presented as frequencies and proportions, while quantitative data following a normal distribution were summarized using the mean and standard deviation to represent central tendency and dispersion. For non-normally distributed quantitative data, the median and interquartile range were used. Spearman’s correlation analysis was conducted to examine the associations among the three dimensions of avian influenza knowledge, attitudes, and practices (KAP). Additionally, a structural equation model (SEM) was developed using Amos 24.0, where the knowledge dimension was treated as an exogenous latent variable, while attitudes and behaviors were considered endogenous latent variables. The structural relationships among these latent variables were established, fitted, and refined, with a significance level set at *α* = 0.05.

## Results

### Sociodemographic characteristics

In this study, a total of 221 valid questionnaires were collected, yielding an effective response rate of 99.55%. Among the respondents, 130 were female (58.8%), and 87 individuals (39.4%) were aged 45–59 years. A total of 33 participants (14.9%) had no formal education, while 193 (87.3%) were married. Additionally, 34 respondents (15.4%) rated their current health status as very good, and 161 (72.9%) reported a monthly income of ≤ 1,000 yuan. Regarding poultry farming, 219 participants (99.1%) were engaged in free-range poultry farming. In terms of poultry types, 136 individuals (61.5%) raised chickens. Additionally, 157 participants (71.0%) spent less than 1 hour per day on poultry-related work. In the past year, 5 respondents (2.3%) had contact with migratory birds, while 27 (12.20%) experienced influenza-like symptoms (Table 1).

**Table 1:** Participant demographics and characteristics.

Variables	Characteristics	Total (%)
Location	Pule Town	121 (54.8)
	Shatian Town	77 (34.8)
	Oujiang Town	23 (10.4)
Gender	Male	91 (41.2)
	Female	130 (58.8)
Age	15~	9 (4.1)
	30~	28 (12.7)
	45~	87 (39.4)
	60~	78 (35.3)
	75 ~ 90	19 (8.6)
Education level	No formal education	33 (14.9)
	Primary school	84 (38.0)
	Junior high school	90 (40.7)
	High school/Technical secondary school	13 (5.9)
	College/Higher vocational College	1 (0.5)
Marital status	Unmarried	6 (2.7)
	Married	193 (87.3)
	Divorced	1 (0.5)
	Widowed	21 (9.5)
Current health status	Excellent	34 (15.4)
	Good	94 (42.5)
	Average	51 (23.1)
	Poor	37 (16.7)
	Very poor	5 (2.3)
Monthly personal income (Yuan)	<1,000	161 (72.9)
	1,000~	40 (18.1)
	3,000~	20 (9.0)
Occupation type	Agricultural (vegetable) market salesperson	1 (0.5)
	Large-scale poultry farmer	1 (0.5)
	Small-scale poultry farmer	219 (99.1)
Type of poultry raised	Chickens	136 (61.5)
	Both chickens and ducks	84 (38.0)
	Other	1 (0.5)
Average daily hours spent on poultry (hours)	<1	157 (71.0)
	1~	60 (27.1)
	3~	4 (1.8)
Years of poultry-related work (years)	<5	40 (18.1)
	5~	22 (10.0)
	10 ~	159 (71.9)
Contact with migratory birds in the past year	Yes	5 (2.3)
	No	207 (93.7)
	Unsure	9 (4.1)
Experienced influenza-like symptoms in the past year	Yes	27 (12.2)
	No	189 (85.5)
	Unsure	5 (2.3)

### Knowledge, attitude, and behavior practices status

#### Overall score of the questionnaire on knowledge, attitude, and practices

**Figure 1:**
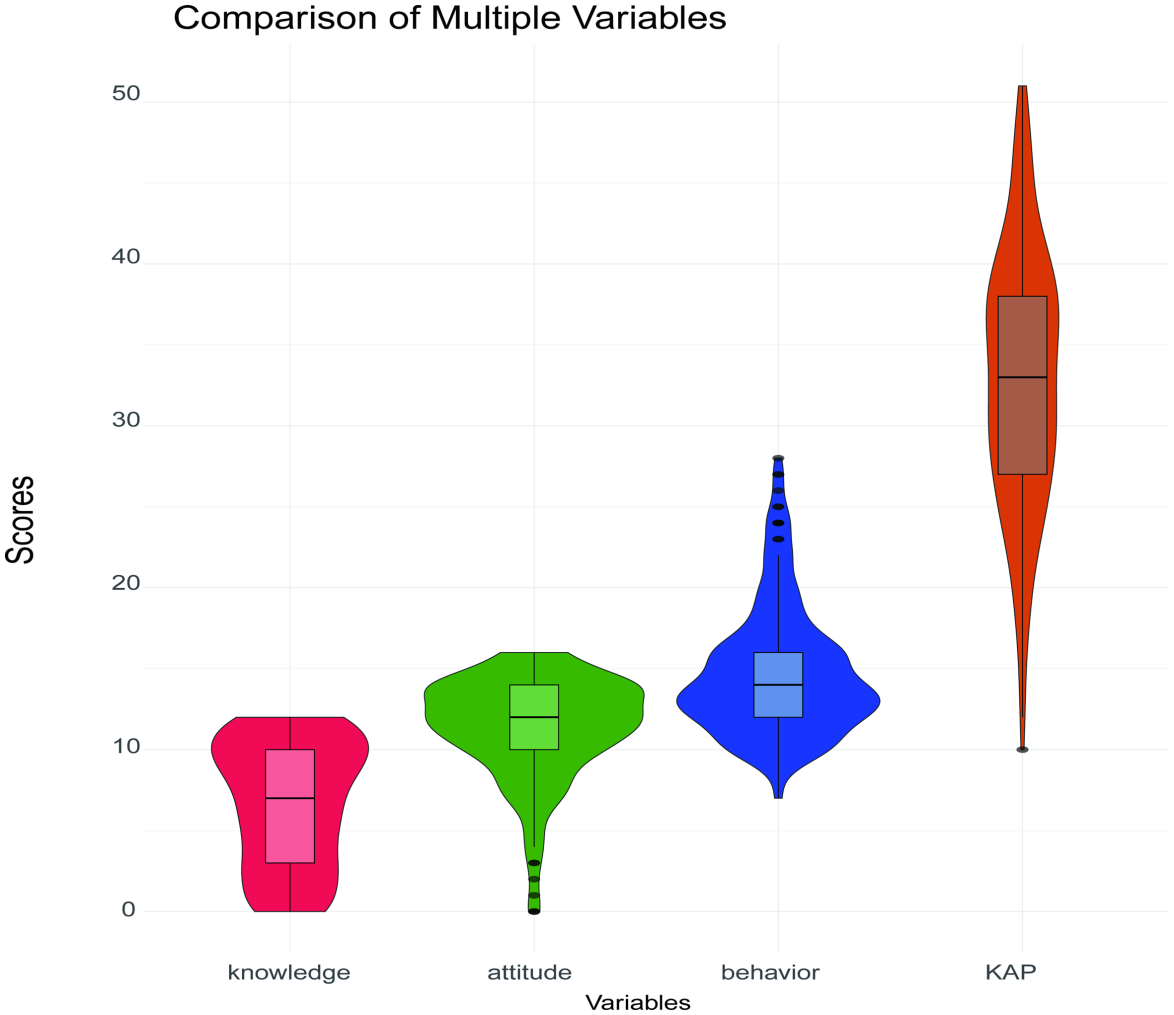
The score distributions of knowledge, attitude, practices and KAP.

In this survey, the overall score range of the questionnaire assessing poultry farmers’ knowledge, attitude, and behavior toward avian influenza around the migratory bird habitat was between 10 and 51 points, with an average score of 32.97 ± 7.95. The overall scoring rate of the questionnaire was 51.51%, which is considered to be at a poor level. Detailed scores for each dimension are provided in Figure 1.

#### Knowledge awareness of avian influenza among poultry farmers around the migratory bird habitat in Guidong County

The highest level of awareness was observed for the statement, “Eating eggs, poultry meat, etc., only after they are thoroughly cooked can prevent human avian influenza,” with an awareness rate of 74.66%. In contrast, the lowest awareness was recorded for the statement, “Fever, cough, and sore throat are early symptoms of avian influenza infection,” with only 30.77% of respondents answering correctly. Additionally, merely 40.27% of participants recognized that avian influenza in humans can be transmitted through contact with migratory birds (Table 2).

**Table 2:** Knowledge awareness of avian influenza among poultry farmers around the migratory bird habitat in Guidong.

Knowledge of avian influenza	Number of people aware	Awareness rate (%)
Fever, cough, and sore throat are early symptoms of avian influenza infection.	68	30.77
Human avian influenza can be transmitted through contact with patients infected with avian influenza.	136	61.54
Human avian influenza can be transmitted through contact with sick or dead chickens, ducks, and other birds.	113	51.11
Human avian influenza can be transmitted through contact with the excretions of infected birds.	107	48.41
Human avian influenza can be transmitted through contact with objects and water contaminated with avian influenza virus.	122	55.20
Human avian influenza can be transmitted through contact with migratory birds.	89	40.27
Human avian influenza can be transmitted through the consumption of chickens, ducks, and other birds.	126	57.01
Sick or dead birds should be buried or incinerated.	164	74.20
Human avian influenza can be transmitted through contact with animals such as pigs and cats.	99	44.80
Thoroughly cooking eggs, poultry meat, and other products before consumption can prevent human avian influenza.	165	74.66
Avoiding contact with waterfowl and migratory birds can prevent human avian influenza.	149	67.42
Avian influenza virus can be transmitted between humans.	146	66.06

#### Attitudes toward avian influenza among poultry farmers around the migratory bird habitat in Guidong County

A total of 85.1% of respondents acknowledged the need to strengthen personal protective measures against avian influenza, and 80.09% expressed a willingness to gain more knowledge about the disease. However, only 12.22% believed they were personally at risk of contracting avian influenza, and just 20.36% considered it likely that the disease could occur in their local area (Table 3).

**Table 3:** Attitudes toward avian influenza among poultry farmers around the migratory bird habitat in Guidong.

Attitudes toward avian influenza	Number of people with a positive attitude	Proportion (%)
Are you concerned about the avian influenza epidemic?	156	70.59
Do you believe that avian influenza could potentially occur in your vicinity?	45	20.36
Do you think you could possibly contract avian influenza?	27	12.22
Do you feel it is necessary to strengthen personal protective measures against avian influenza?	188	85.07
Are you willing to learn more about avian influenza?	177	80.09
Would you be willing to disseminate information about avian influenza to others?	175	79.18
If domestic birds in your household become ill, would you be willing to inform the epidemic prevention department?	125	56.56
Are you willing to receive the avian influenza vaccine?	168	76.02

#### Behavioral practices regarding avian influenza among poultry farmers around the migratory bird habitat in Guidong County

A total of 77.82% of participants reported that they consistently ventilate poultry houses. However, only 7.69, 3.62, 4.52, and 9.50% indicated that they adopt personal protective measures such as wearing a uniform/apron, a mask, gloves, and boots/waterproof shoes, respectively, when handling live poultry (Table 4).

**Table 4:** Behavioral practices situation of avian influenza among poultry farmers around the migratory bird habitat in Guidong.

Avian influenza prevention behaviors	Always (%)	Often (%)	Rarely (%)	Never (%)
Do you regularly ventilate the poultry house?	172 (77.82)	33 (14.93)	13 (5.88)	3 (1.36)
Do you clean the cages periodically?	92 (41.63)	74 (33.48)	39 (17.65)	16 (7.24)
Do you disinfect the cages on a regular basis?	31 (14.03)	40 (18.10)	53 (23.98)	97 (43.89)
Do you wear a work uniform/apron when handling live birds?	17 (7.69)	13 (5.88)	34 (15.38)	157 (71.04)
Do you wear a mask when in contact with live birds?	8 (3.62)	14 (6.33)	25 (11.31)	174 (78.73)
Do you wear gloves when handling live birds?	10 (4.52)	13 (5.88)	30 (13.57)	168 (76.02)
Do you wear boots/waterproof shoes when interacting with live birds?	21 (9.50)	21 (9.50)	44 (19.90)	135 (61.08)

#### Correlation analysis of knowledge, attitudes, and practices related to avian influenza

**Figure 2:**
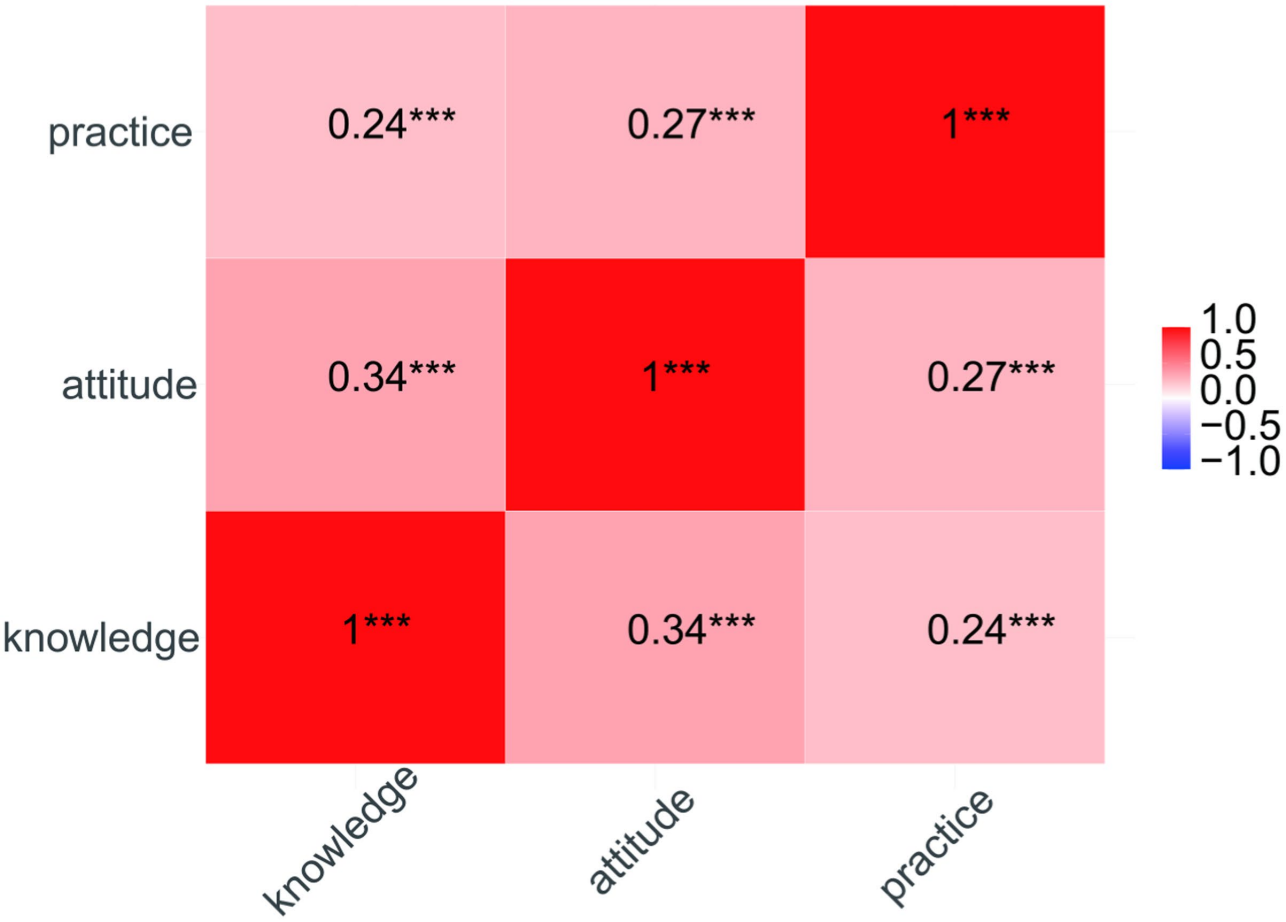
The correlation of knowledge, attitude, and practice among poultry farmers around the migratory bird sites in Guidong. ***p < 0.01.

Spearman correlation analysis revealed statistically significant positive correlations among the knowledge, attitude, and practice scores of poultry farmers regarding avian influenza in the migratory bird habitat of Guidong County (*p* < 0.01) (Figure 2).

### Construction and validation of the structural equation model

An initial structural equation model (SEM) was developed using *AMOS 24.0*, based on the following hypotheses: *H1*: Knowledge of avian influenza directly influences prevention and control behaviors; *H2*: Attitudes toward avian influenza directly influence prevention and control behaviors; *H3*: Knowledge indirectly influences prevention and control behaviors through attitudes.

The model included three latent variables: Avian influenza knowledge (exogenous), with 12 observed variables. Attitudes and prevention and control behaviors (endogenous), with 8 and 7 observed variables, respectively. Confirmatory factor analysis led to the removal of items 1, 9, 10, 11, and 12 from the knowledge dimension; items 2, 3, 4, and 12 from the behavior dimension; and items 5, 6, 7, 10, and 11 from the attitude dimension. To improve model fit, the residuals e11 and e17 were correlated based on the Modification Indices. Variable assignments for both latent and observed variables are presented in Table 5.

**Table 5:** Latent variables and measured variables assignment table.

Latent variables	Measured variables
Knowledge	Human avian influenza can be transmitted through contact with dead or sick chickens and ducks (b2)
	Human avian influenza can be transmitted through contact with the excretions of sick birds (b3)
	Human avian influenza can be transmitted through contact with items and water contaminated with avian influenza virus (b4)
	Human avian influenza can be transmitted through contact with patients suffering from avian influenza (b5)
	Human avian influenza can be transmitted through contact with migratory birds (b6)
	Human avian influenza can be transmitted through contact with animals such as pigs and cats (b7)
	Human avian influenza can be transmitted through consumption of chickens, ducks, and other birds (b8)
Attitude	Do you care about the avian influenza epidemic? (d4)
	Are you willing to learn more about avian influenza? (d8)
	Are you willing to spread information about avian influenza to others? (d9)
Behavior	Do you wear a work uniform/apron when handling live birds? (c10)
	Do you wear a mask when handling live birds? (c11)
	Do you wear boots/waterproof shoes when handling live birds? (c13)

**Figure 3:**
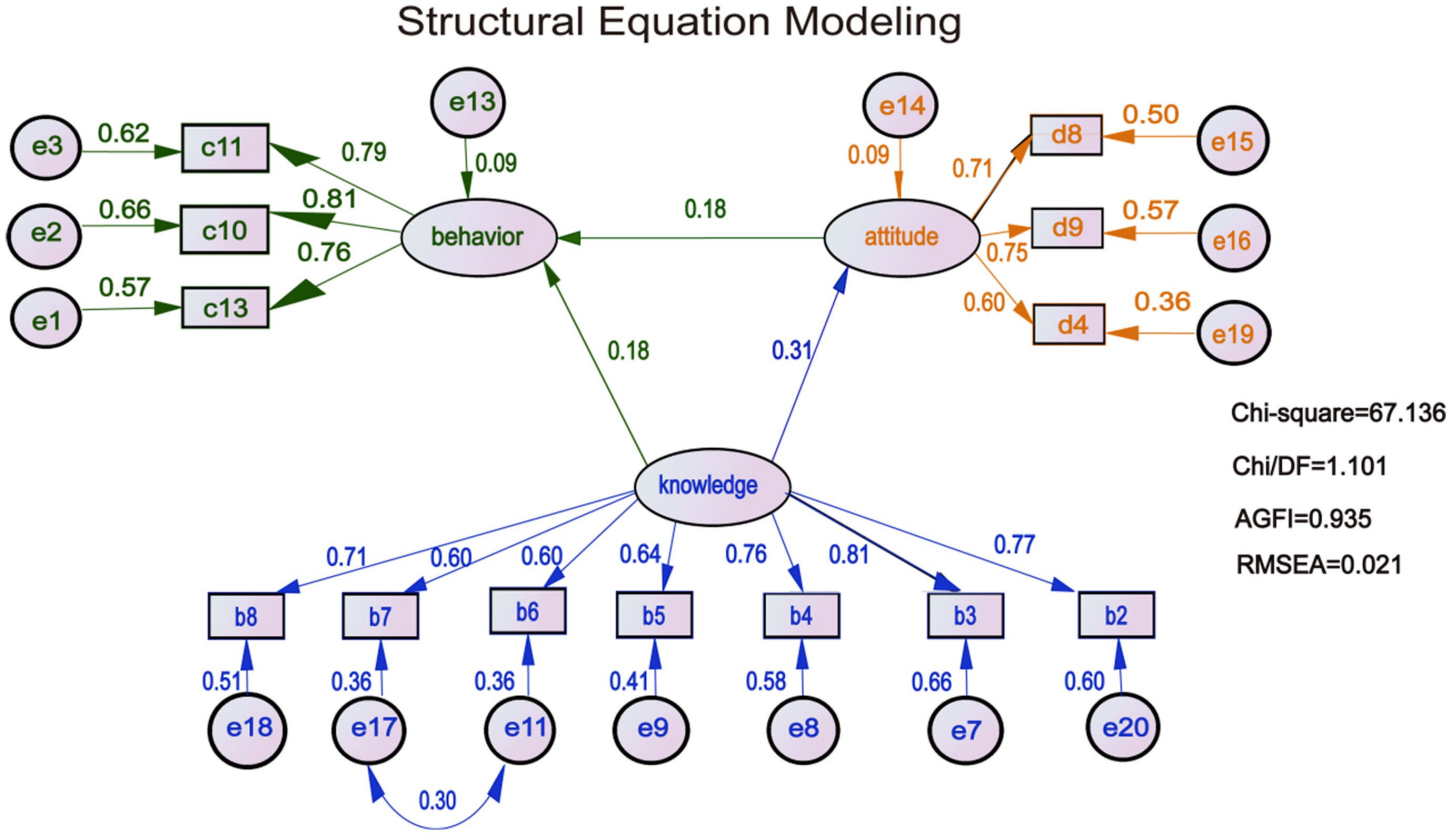
The final SEM. Rectangle shows observed variables, ellipses indicate potential variables, and circles represent residual terms. The values of single-headed arrows represent the standardized coefficients. All paths were significant (*p* < 0.05).

The revised and optimized *SEM* for avian influenza KAP among poultry farmers in Guidong County’s migratory bird habitat is illustrated in Figure 3.

### Model fit and path analysis

The final model demonstrated a good fit with the following indices: *Chi/DF* = 1.101, *GFI* = 0.956, *AGFI* = 0.935, *RMR* = 0.024, and *RMSEA* = 0.021, all within acceptable thresholds (Table 6).

**Table 6:** The fit indices of structural equation model (SEM).

Fit index	*CMIN/DF*	*GFI*	*AGFI*	*CFI*	*RMR*	*RMSEA*
Reference index	<3	>0.9	>0.9	>0.9	<0.05	<0.05
Final model index	1.101	0.956	0.935	0.994	0.024	0.021

Path analysis supported all three proposed hypotheses (*p* < 0.05). Knowledge of avian influenza had a direct positive effect on prevention and control behaviors, with a standardized effect size of 0.183. It also had an indirect positive effect via attitudes, with an effect size of 0.056. The total effect of knowledge on behavior was 0.239. Additionally, attitudes had a direct positive effect on prevention and control behaviors, with a standardized effect size of 0.181 (Table 7).

**Table 7:** Hypothesis testing results for path coefficients of knowledge, attitude and practice.

Statements	Unstandardized estimates	Standardized estimates	S. E.	*T*-value	*p*-value	Label
Knowledge → attitude	0.204	0.306	0.057	3.589	<0.001	H1
Attitude → practice	0.291	0.181	0.146	1.992	0.046	H2
Knowledge → practice	0.196	0.183	0.089	2.197	0.028	H3

A *p*-value < 0.05 was considered statistically significant.

## Discussion

In this study, we conducted the first systematic KAP assessment among poultry farmers in Guidong County—an ecologically vulnerable node along central China’s migratory bird flyway. The complex relationships among knowledge, attitudes, and preventive behaviors were analyzed. The findings revealed that the overall KAP score was only 51.51%, indicating a poor level. Specifically, the knowledge score was 57.20%, the attitude score 52.58%, and the behavior score merely 47.42%. These low scores may be attributed to the underdeveloped economy of Guidong County and its insufficient investment in public health infrastructure. Migratory birds are known vectors for the transregional spread of avian influenza viruses, such as H5N1 and H7N9 subtypes (26). Their frequent presence significantly increases the exposure risk for local poultry (27). While our observational design precludes causal claims, the observed deficiencies in both awareness and protective behaviors among farmers suggest a potential transmission chain of “migratory birds – domestic poultry – humans.” These insights are crucial for optimizing prevention and control strategies in high-risk areas.

Although a majority of participants (74.2%) were aware of the proper disposal methods for sick or dead birds, only 30.8% recognized early avian influenza symptoms, and 66.1% overestimated human-to-human transmission risk. These figures exceed those reported in a similar study from India (28). Such gaps in knowledge pose dual threats: delayed symptom recognition may hinder early detection and isolation, while exaggerated fears of human transmission could provoke irrational responses, such as the misuse of antibiotics. Therefore, in migratory bird regions, health education should emphasize the cognitive chain of “symptom recognition — timely reporting — scientific disposal.”

The significant disparity between attitude and behavior scores highlights a deeper issue: while 85.07% of participants acknowledged the importance of protective measures, only a small fraction reported using personal protective equipment (PPE) such as masks and gloves when handling poultry. This aligns with findings by Ayim-Akonor et al. (19). Evidence shows that using personal protective equipment (PPE) such as masks and gloves reduces avian influenza virus transmission (29). Yet, only 12.22% of participants perceived themselves at risk, indicating a “knowing-but-not-doing” phenomenon (30). This discrepancy may be explained through the lens of the Health Belief Model (HBM), where low perceived susceptibility (“I am unlikely to get infected”) and perceived barriers (e.g., cost or discomfort of PPE use) outweigh perceived benefits (31). Misunderstandings about avian influenza may result in inappropriate behaviors, undermining disease prevention efforts and potentially exacerbating risks (12). Hence, future health education should not only focus on disseminating accurate information but also improve risk perception, practical skills, and rectify flawed attitudes.

Structural equation modeling demonstrated that both knowledge and attitudes were associated with preventive behaviors toward avian influenza. Previous studies have confirmed that individuals with higher levels of awareness are more likely to adopt appropriate preventive attitudes and behaviors (32) In line with the KAP framework, insufficient knowledge, low perceived risk, and inadequate practices collectively contribute to increased infection risk (15). Knowledge influences behavior indirectly via attitude, and this indirect effect was found to be stronger than the direct effect. However, as this cross-sectional study cannot establish temporal precedence, alternative interpretations remain plausible. As a necessary condition for behavioral change, knowledge must be coupled with favorable attitudes to foster practice improvement (22, 33). Therefore, avian influenza health education should emphasize not only knowledge dissemination but also attitudinal transformation to bridge the gap between awareness and action.

Our study was conducted during a distinctive phase of China’s COVID-19 pandemic management. The research period (July 2021) coincided with a time when nationwide pandemic control measures had been fully lifted, resulting in minimal COVID-19-related restrictions. This operational context allowed uninterrupted implementation of household surveys along migratory bird routes, where investigators conducted door-to-door questionnaires with poultry farmers. A key strength of this study lies in its systematic spatial coverage of farming communities adjacent to migratory pathways, which reduced selection bias. Furthermore, this research represents the first KAP investigation explicitly linking avian biogeography (e.g., Central Asian Flyway dynamics) with human behavioral determinants—an underexplored interface in poultry worker studies.

This study has several limitations. Due to its cross-sectional design, causal relationships among knowledge, attitudes, and practices cannot be firmly established. For instance, inadequate protective behavior may demotivate individuals from acquiring relevant knowledge. Future studies should consider a longitudinal design with intervention-control components to evaluate the effectiveness of health education. Second, while our survey encompassed most poultry farmers along migratory routes, some individuals were unavailable during data collection. This convenience sampling approach may have led to an overestimation of the population’s KAP levels. It is recommended that future research expand the sampling scope and incorporate contextual environmental variables such as migratory bird activity and poultry density to construct a multi-level risk prediction model. Finally, given the study’s timing during pandemic normalization, subsequent research should track behavioral changes across post-COVID recovery phases to clarify lasting impacts on biosecurity practices, particularly regarding sustained mask usage and risk perception evolution.

In conclusion, poultry farmers in Guidong County’s migratory bird habitats exhibit suboptimal KAP levels regarding avian influenza. Accurate knowledge and a positive attitude are crucial for improving farmers’ preventive behaviors. Based on the ecological characteristics of migratory birds, and the study population predominantly comprised individuals aged 45 years and older, with most having attained junior high school education or lower, a “three-in-one” intervention strategy is proposed: (1) Knowledge Enhancement: Develop dialect-specific multimedia educational materials in local dialects focusing on symptom recognition and common misconceptions; (2) Behavior Promotion: Provide governmental subsidies for PPE and include masks and gloves in essential farming supplies, coupled with training programs to improve protective awareness and skills; (3) Institutional Support: Establish a joint prevention mechanism including “risk alerts during migratory seasons – routine disinfection – behavioral monitoring,” with implementation responsibilities delegated to village committees. This integrated model may not only reduce zoonotic transmission risks but also enhance biosecurity in farming and support rural revitalization initiatives.

## Data Availability

The raw data supporting the conclusions of this article will be made available by the authors, without undue reservation.
